# An Improved Method for Estimating Chromosomal Line Origin in QTL Analysis of Crosses Between Outbred Lines

**DOI:** 10.1534/g3.111.000109

**Published:** 2011-06-01

**Authors:** Lucy Crooks, Carl Nettelblad, Örjan Carlborg

**Affiliations:** * Department of Cell and Molecular Biology, Uppsala University, SE-751 24 Uppsala, Sweden; § Department of Information Technology, Uppsala University, SE-751 05 Uppsala, Sweden; ** Department of Animal Breeding and Genetics, Swedish University of Agricultural Sciences, SE-750 07 Uppsala, Sweden

**Keywords:** interval mapping, outbred line cross, line origin probabilities, hidden Markov model, SNP chip

## Abstract

Estimating the line origin of chromosomal sections from marker genotypes is a vital step in quantitative trait loci analyses of outbred line crosses. The original, and most commonly used, algorithm can only handle moderate numbers of partially informative markers. The advent of high-density genotyping with SNP chips motivates a new method because the generic sets of markers on SNP chips typically result in long stretches of partially informative markers. We validated a new method for inferring line origin, triM (tracing inheritance with Markov models), with simulated data. A realistic pattern of marker information was achieved by replicating the linkage disequilibrium from an existing chicken intercross. There were approximately 1500 SNP markers and 800 F_2_ individuals. The performance of triM was compared to GridQTL, which uses a variant of the original algorithm but modified for larger datasets. triM estimated the line origin with an average error of 2%, was 10% more accurate than GridQTL, considerably faster, and better at inferring positions of recombination. GridQTL could not analyze all simulated replicates and did not estimate line origin for around a third of individuals at many positions. The study shows that triM has computational benefits and improved estimation over available algorithms and is valuable for analyzing the large datasets that will be standard in future.

Analysis of experimental line crosses using genetic markers has been successful in identifying quantitative trait loci (QTL) for a wide range of physical, behavioral, and disease traits in plants, model organisms, and livestock species (Corva and Medrano 2001; Mackay 2001; Maloof 2003; Hocking 2005; Rothschild *et al.* 2007). Ultimate aims of QTL analyses include determining the number of genes influencing a trait and the nature and size of their effects, identification of important pathways in disease, and discovery of genes that may be useful in breeding programs. Resolving QTL to the level of the causative gene has been challenging, although there are some notable examples: *IGF2* affecting muscle growth in pigs (Van Laere *et al.* 2003), *ORFX* for fruit size in tomatoes (Frary *et al.* 2000), and *achaete-scute* influencing bristle number in *Drosophila melanogaster* (Long *et al.* 2000). An obstacle to identifying the underlying genes has been the relatively large size of QTL regions, which can encompass hundreds of genes. For line crosses, ultimately the lower limit on the size of QTL regions is set by the number of recombinations that have occurred in the pedigree. However, to achieve the best resolution and statistical power within this constraint, it is important to extract the maximal possible information from the genetic markers.

In linkage-based QTL analyses, such as with line crosses, marker genotypes are used to infer the inheritance of chromosomal segments through the pedigree. For line crosses, the key factor is the line origin of segments (*i.e.* whether they are inherited from a founder individual of line 1, or a founder individual of line 2). Most analyses apply interval mapping (Lander and Botstein 1989) where an association between line origin and phenotype is tested at regularly spaced positions along the genome. Many of these positions will not coincide with markers that unambiguously indicate the line origin (fully informative markers), in which case the probability of having each line origin is estimated based on the genotypes of available markers and the expected frequency of recombination between these markers and the test position. With inbred lines, all selected markers are fully informative, and only the genotypes of the two markers flanking the test position are needed. For outbred lines, however, many markers are likely to be only partially informative (more than one line origin is compatible with the genotype although at least one line origin can be excluded) because the same marker alleles are segregating in both lines. Haley *et al.* (1994) showed that for outbred lines, the genotypes of all partially informative markers up to the nearest fully informative marker on each side should be considered. They proposed an algorithm that separately evaluates the probability of each possible combination of line origins between fully informative markers, then sums and normalizes the results. To date, this algorithm, or variants of it, has been the most commonly used method for estimating line origin probabilities in outbred line crosses.

The use of SNPs has become standard in QTL mapping, and because of their high frequency in the genome, the number of scored markers has increased considerably from older technologies. A recent advance is the SNP chip, which allows parallel genotyping of hundreds of thousands of selected SNPs. These SNPs are typically chosen from a few populations of interest. As a result, some will not be fully informative in studies involving other populations. However, because so many SNPs are included, there will still be many that are at least partially informative. The advantage of the chips is that they remove the need for time-consuming development, selection, and testing of markers for each study and provide a standard set of markers with known location, allowing direct comparisons between studies. Chips of at least 50,000 SNPs are currently available for cow, pig, dog, chicken, sheep, mouse, and maize, for example. The Haley *et al.* (1994) algorithm worked well for the small numbers of markers that were used at the time. However, the maximum number of calculations required by the algorithm scales exponentially with the number of partially informative markers in stretches with no intervening fully informative markers. On most computers, the algorithm crashes when there are more than 20 partially informative markers in such stretches (Nettelblad *et al.* 2009). Therefore, it is not suitable for handling SNP chip datasets. To utilize all the information available from SNP chips, a new method is needed.

We recently developed a new tool for estimating line origin probabilities. The algorithm scales logarithmically with the number of markers included, making analysis of very large datasets feasible. We have designated this method triM (tracing inheritance with Markov models), which is implemented in the existing codebase cnF2freq (Nettelblad *et al.* 2009). Here we evaluate the performance of triM on simulated datasets of approximately 1500 SNPs of mixed information content. Although the original Haley *et al.* (1994) algorithm cannot deal with such datasets, they can be analyzed if the algorithm is modified to include information from only a subset of markers. We compare the behavior of triM to one such modified version of the Haley *et al.* (1994) algorithm incorporated in the web-based tool GridQTL (Seaton *et al.* 2006). The size of these datasets is at the reported upper limit for GridQTL, but we emphasize that triM can easily analyze much larger datasets than this.

## Methods

### triM

triM employs an algorithm developed for hidden Markov models (Rabiner 1989). Using these models to track the parental origin of alleles in linkage mapping was introduced by Lander and Green (1987). Specific application of hidden Markov models to QTL analysis in intercrosses was implemented in R/QTL (Broman *et al.* 2003) but limited to the cases of inbred or haplotyped data. The underlying genetic model assumed by triM is the same as for the Haley *et al.* (1994) algorithm, but the advance in triM is full exploitation of the Markov property. Results from triM are expected to be identical to those that would be obtained from the Haley *et al.* (1994) algorithm, were the calculations possible.

**Figure 1:**
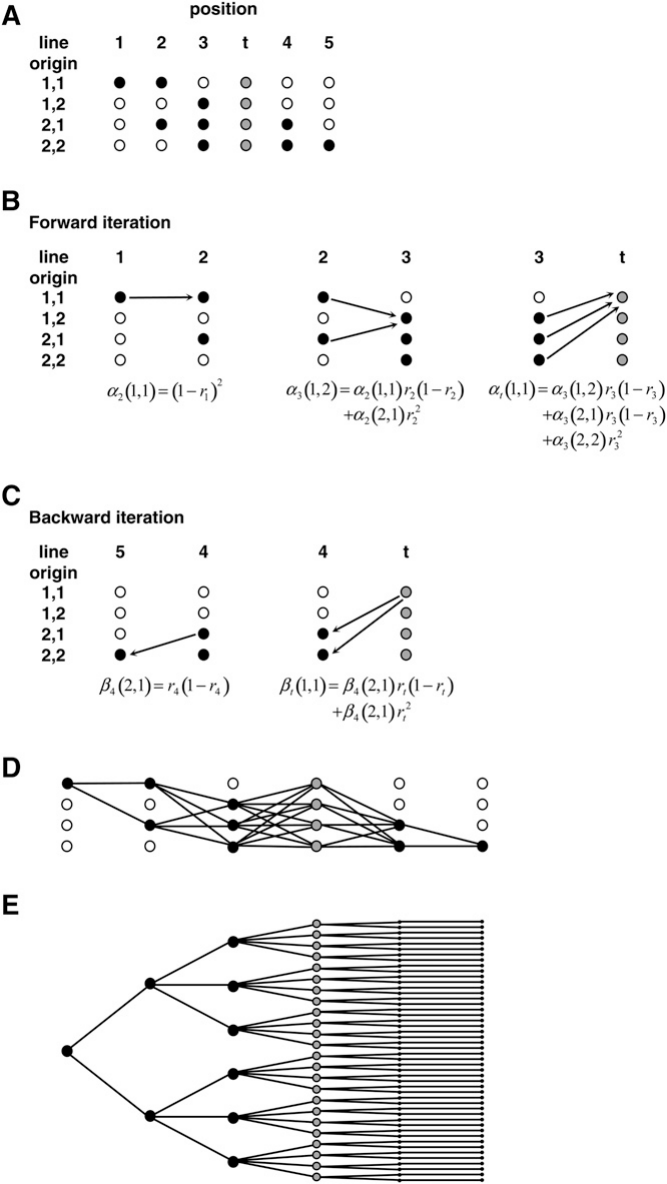
Outline of the forward-backward algorithm used in triM. (A) Schematic of line origin states along a section of chromosome flanked by two fully informative markers. Positions 1-5 are markers, and *t* is the position being tested as a QTL. Circles represent the possible line origins, where *i*,*j* symbolizes that the maternal haplotype originates from line *i* and the paternal haplotype from line *j*. Black filled circles show line origins that are compatible with the marker genotypes and pedigree. At *t*, all line origins are treated as compatible, shown by the gray filled circles. (B) Calculation of $\alpha_{p}\left(i,j\right)$, the combined probability of having line origin *i*,*j* at position *p* and the observed marker genotypes from positions 1 to *p*, in the forward step. *r_p_* is the recombination frequency between position *p* and the next position. Only $\alpha_{p-1}$ and *r_p−1_* are needed in each iteration. For clarity, calculations are only shown for one line origin. Although only compatible line origins are included in the example, triM considers incompatible origins with a low probability to allow for genotyping errors. (C) Calculation of $\beta_{p}\left(i,j\right)$, the probability of having the observed marker genotypes from position *p* +*1* to the end, given that the line origin is *i*,*j* at position *p*, in the backward step. Only $\beta_{p-1}$ and *r_p_* are needed in each iteration. Again, calculations are shown for only one line origin. Having obtained $\alpha_{p}$ and $\beta_{p}$, the probability of line origin *i*,*j* at the test position is given by $\alpha_{t}\left(i,j\right)\beta_{t}\left(i,j\right)/\sum_{k,l}\alpha_{t}\left(k,l\right)\beta_{t}\left(k,l\right)$. (D) Representation of the number of operations needed for the forward-backward algorithm in this example. (E) For comparison, a representation of the number of operations required under the original algorithm for the same example.

A Markov model consists of a series of states where the state at any point depends only on the immediately preceding state and not any of the states before that. Line origin along a chromosome is a Markov process if the frequencies of recombination in each interval are independent of each other. triM therefore applies Haldane’s mapping function, which fulfills the Markov property by assuming no interference between crossovers. The extension to a hidden Markov model is that the states themselves are not observable but influence the values of a second variable, which is observed. In this context, the state is the line origin and the output variable is the marker genotype. The line origin probability at a test position is calculated by iteratively building up the probability of each line origin at successive marker positions, working forward and backward from both ends of the chromosome. For each marker position, only the probabilities of moving from the possible line origins at the preceding marker are needed; all the previous transition probabilities are contained in the line origin probabilities at the preceding marker. The calculation therefore collapses back to a limited number of operations at each marker, which is the basis of its efficiency. The forward and backward probabilities for each test position are multiplied together and normalized by the product summed over all origins. The principle of the algorithm is illustrated in Figure 1, and additional details are given in Nettelblad *et al.* (2009). triM is available for download at http://www.computationalgenetics.se under “Software.” We are currently developing a software package in the statistical environment R, incorporating triM, that will perform the complete process of QTL analysis for crosses between outbred lines.

### Simulation

To evaluate triM with a realistic pattern of marker information, the simulation was based on data from a three-generation chicken intercross (Kerje *et al.* 2003) where dense SNP genotypes were available for two families (Groenen *et al.* 2009). These families originated from one Red Junglefowl male and three White Leghorn females and contained 23 and 25 F_2_ individuals, respectively. We simulated a single chromosome using the data from chromosome 1. Markers with missing genotypes in the parents or grandparents were removed. Some additional markers were also removed because of inheritance errors or uncertainty in their order, and a small number of genotype corrections were made, leaving a total of 1508 markers covering 451 cM. Genetic positions of markers were taken from the sex-averaged consensus linkage map (Groenen *et al.* 2009) where possible. The linkage positions were not used in a few cases where the order of one or two markers differed between the linkage map and the genome assembly. Remaining genetic positions were estimated from the nearest flanking markers in the linkage map, assuming a linear relationship between genetic and physical positions over the interval. Markers that had the same genetic position *x* were separated by spacing them evenly from *x*-0.05 to *x*+0.049 cM. The average distance between markers was 0.3 cM. The biggest gap between markers was 8 cM, between the second and third, and the third and fourth markers. There were 28 gaps of 1-4 cM; the remaining distances between markers were less than 1 cM.

The pattern of marker information in an F_2_ individual depends on both the genotypes of the grandparents and how often successive markers that are heterozygous in a grandparent or parent, are heterozygous in their offspring. Essentially, it is determined by which alleles occur together on the same copy of a chromosome (the haplotypes) in the grandparents. It was not possible to unambiguously haplotype the grandparents because they had too few genotyped offspring. Therefore, we instead haplotyped the parents, and treated the haplotype transmitted to the parent as one haplotype in the grandparent, with the remaining alleles forming the second haplotype. When there were several parents that could be haplotyped for a given grandparent, one was chosen at random. Haplotyping was performed by the following procedure. First the allele originating from each parent was determined for the straightforward cases of markers that were homozygous or heterozygous with at least one parent homozygous. For the other heterozygous markers, the parental origin was inferred by looking at the combination of F_2_ genotypes at the marker and a second heterozygous marker where the origin was known. The second marker was chosen so that the set of F_2_ genotypes would differ according to which alleles were inherited together from the same parent.

Genotypes of parents and then F_2_ individuals were simulated from the grandparent haplotypes. The number of crossovers was sampled from a Poisson distribution with mean equal to the chromosome length in Morgans. The positions of each crossover were then sampled from a uniform distribution covering the length of the chromosome. A starting haplotype was chosen at random. Moving along the chromosome, alleles were taken from this haplotype until a recombination position was reached. Then alleles were taken from the second haplotype. This continued, with the sampled haplotype changing after each crossover, to the end of the chromosome. Individuals were generated using the pedigree structure of the original three-generation intercross (Kerje *et al.* 2003). There were four male and 37 female parents and 773 F_2_ individuals.

## Analysis

Line origin probabilities were estimated at 1-cM intervals. Both methods estimate four probabilities for each F_2_ individual and position, the product of two possible line origins for each haplotype. triM was used to obtain probabilities for 1000 replicates of the complete dataset. Attempts were then made to analyze the data using GridQTL, version 1.4.1. We were unable to obtain results from GridQTL for several tested replicates although the online documentation stated that GridQTL could handle around this number of markers. Therefore, we experimented with datasets of reduced size, produced by successively removing the last 100 markers. With 1200 markers, GridQTL produced results for nine of the first 10 replicates. Because of the difficulties in obtaining results from GridQTL, we compared triM and GridQTL for these nine replicates. To avoid possible artifacts at the end of the chromosome, as triM estimates were based on all 1508 markers, we compared only the region covered by 1150 markers, excluding the last 50 markers available to GridQTL, which was 333 cM. For many individuals, there were regions where GridQTL reported all line origin probabilities as zero (*i.e.* where probability estimates were missing). Even though these are the cases that have the largest impact on the power and precision for subsequent QTL analyses, we have not included these probabilities in our comparison. Only data points for which there were estimates from both GridQTL and triM were compared. Both methods were timed. For GridQTL, timing estimates were less accurate, because the viewer window in GridQTL had to be manually refreshed to check if an analysis had finished. The following two aspects of the results were considered.

### Line origin error

From the probabilities estimated by each method, we calculated the overall probabilities that each haplotype originates from line 1. The probabilities given by triM are, in order, (i) both haplotypes originate from line 1, (ii) the maternal haplotype originates from line 1 and the paternal haplotype from line 2, (iii) the maternal haplotype originates from line 2 and the paternal haplotype from line 1, and (iv) both haplotypes originate from line 2. In the results from GridQTL, the order of probabilities (ii) and (iii) are switched. Probabilities (i) and (ii) were summed for the maternal haplotype, and (i) and (iii) were summed for the paternal haplotype. The true line origins are specified in the simulation. For each haplotype, we define the line origin error at position *i*, e(i), as

$$e\left(i\right)=1-p_{1}\left(i\right)\text{ when the true origin is line 1}$$,

$$e\left(i\right)=p_{1}\left(i\right)\text{ when the true origin is line 2}$$,

where $p_{1}\left(i\right)$ is the probability of line 1 origin at position *i*. The average and standard deviation in line origin error was calculated over both haplotypes for all individuals.

### Positions of recombination

Inferred recombination events in the parents can be seen as changes in haplotype line origin probabilities from close to zero to nearly one and vice versa. Only such switches that covered the range of probabilities from 0.025-0.975 were considered; the positions where the probabilities exceeded 0.975 and fell below 0.025 were called the end points of the switch. Switches from GridQTL were not included if there were missing probability estimates within the switch. We defined two summary measures: imprecision and inaccuracy. Imprecision in an estimated recombination position is the distance between the end points of a switch. It quantifies the amount of uncertainty in the estimated position; high imprecision means the method locates a broad region in which recombination has occurred, low imprecision that an interval or short stretch has been pinpointed. Inaccuracy in an estimated recombination position is the distance between the true recombination position and where the line origin probabilities cross 0.5. Occasionally, several consecutive positions had a probability of 0.5; in these cases, the distance to the central position was used. The minimum possible imprecision is 1 cM; the minimum inaccuracy is 0 cM. Most of the largest line origin errors are likely to be where recombination positions have been estimated with high imprecision or inaccuracy.

## Results

**Figure 2:**
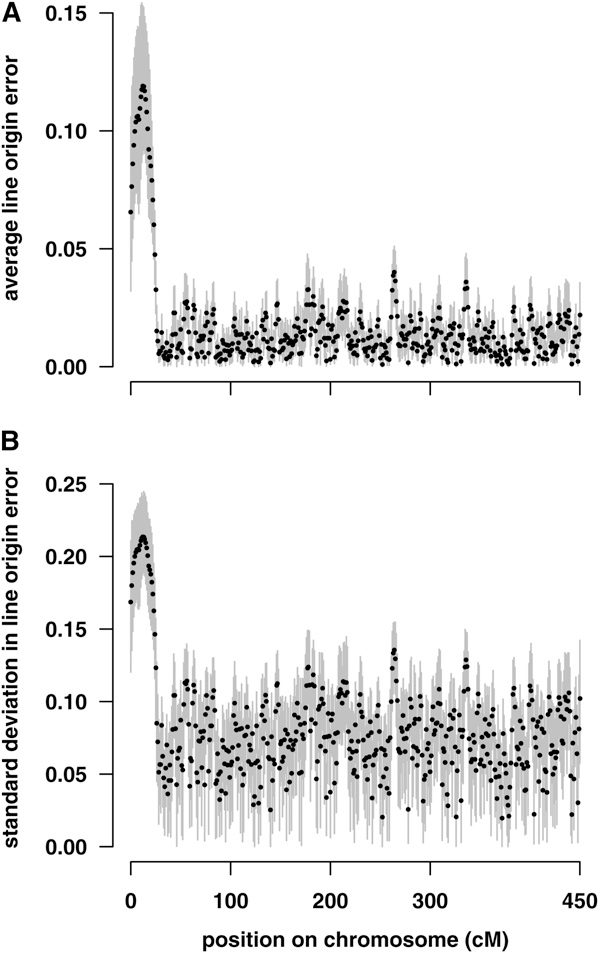
Line origin error for triM. (A) Average line origin error at each position over both haplotypes and all individuals. Dots show the average for 1000 simulation replicates, and gray bars indicate the minimum and maximum average line origin error obtained for a single replicate. (B) Standard deviation in line origin error at each position. Dots show the average standard deviation for 1000 simulation replicates, and gray bars indicate the minimum and maximum standard deviations obtained for a single replicate.

The average line origin error for triM over 1000 simulation replicates ranged from 0.0009 to 0.04 for most of the simulated chromosome (Figure 2A). The error was higher at the start of the chromosome, where the distance between the markers was largest, reaching an average of 0.12 at 12 cM. This position is halfway between one of the pairs of markers that are separated by 8 cM, with the second marker being homozygous for the same allele in all four grandparents, and the low marker information limits how accurately line origin can be estimated. The average line origin error across all positions was 0.017. The actual haplotype line origin corresponds to a probability of either 1 or 0; therefore, the average error is 2% of the range of possible values. The average standard deviation in line origin error within a replicate was 0.08 (Figure 2B). As with the average error, the average standard deviation in the error was higher at the start of the chromosome.

**Figure 3:**
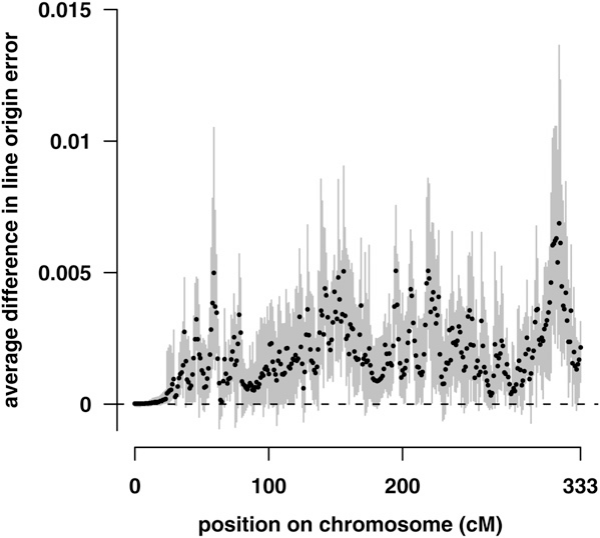
Average difference in line origin error at each position between GridQTL and triM. Only values from triM that were also estimated by GridQTL are included. The end of the chromosome was excluded to avoid artifacts due to the additional marker information used by triM. Positive values mean that the average line origin error was higher for GridQTL than triM; dashed line indicates zero. Dots show averages for nine replicates. Gray bars indicate the minimum and maximum difference in line origin error obtained for a single replicate.

GridQTL had a higher average line origin error over nine replicates than triM at all positions (Figure 3). The difference varied from 0.00001 to 0.007, with an average across all positions of 0.0019. This is equivalent to a 10% higher average error for GridQTL than triM.

**Figure 4:**
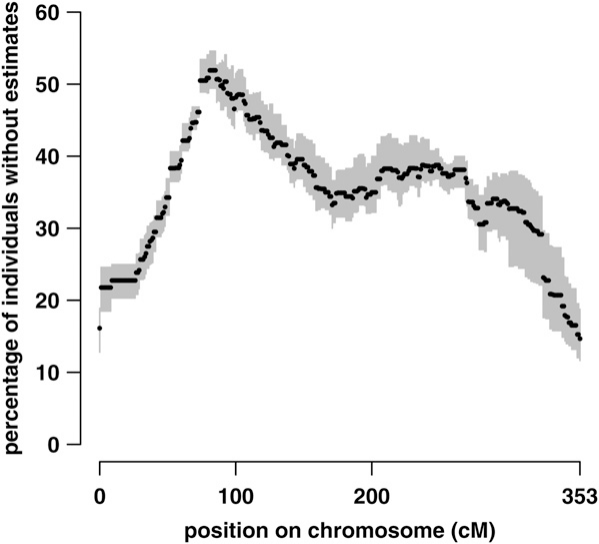
Missing probability values at each position from GridQTL. The percentage of individuals given zero probabilities for all four line origins is shown. Dots show the average for nine replicates. Gray bars indicate the minimum and maximum percentage of individuals with missing values for a single replicate.

The percentage of individuals missing probability estimates from GridQTL at each position is shown in Figure 4. It was between 15 and 52%, with an average over all positions of 35%. For every replicate, over 30% of individuals had missing probabilities in at least 2/3 of the positions.

The analysis times for triM and GridQTL are shown in Table 1. triM completed each analyses in seconds whereas GridQTL took several hours. Table 1 also shows the number of replicates that GridQTL was able to analyze for different numbers of markers.

**Table 1:** Timing comparisons for triM and GridQTL

Number of Markers	GridQTL			triM
	Number of Replicates Completed	Number Timed	Average Time (range) (hr:min)	Average Time (range) (sec)
1508	7	2	9:10 (7:20–11:00)	45 (43–47)
1400	6	3	4:39 (4:35–4:41)	
1300	7	4	3:47 (3:13–4:16)	
1200	9	9	2:33 (2:02–2:58)	

Methods were tested on 10 replicates. Smaller datasets were produced by successively removing the last 100 markers. triM was able to analyze all replicates of the complete dataset with 1508 markers. GridQTL results are from version 1.4.1, running on the ECDF grid in March and April 2010. triM was run on a machine with a 2.66 GHz Intel Core i7 CPU. The current version of the cnF2freq codebase was used with OpenMP support disabled, compiled with gcc 4.2.1 and no specifically tuned optimization flags.

**Figure 5:**
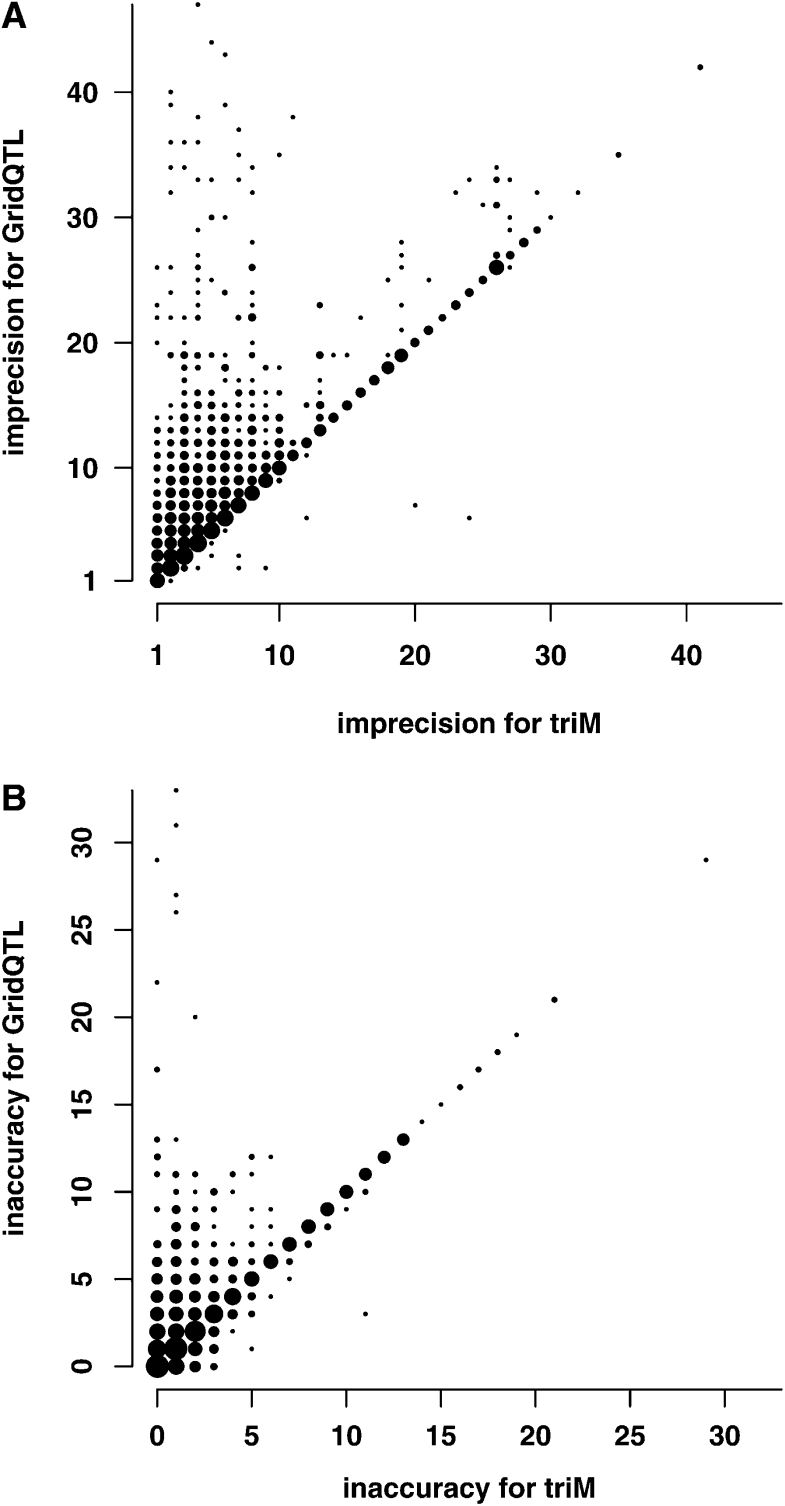
Estimation of recombination positions by triM and GridQTL. Imprecision and inaccuracy were measured as described in the METHODS. The size of the circles is in proportion to the number of data points at each pair of values. Points above the diagonal represent cases where imprecision or inaccuracy in the estimated position of recombination was greater for GridQTL than for triM; points below the diagonal, cases where imprecision or inaccuracy was greater for triM than GridQTL; and points on the diagonal, cases where imprecision or inaccuracy was the same for both methods. The further the points are from the diagonal, the bigger the difference between the results. (A) Imprecision in estimated positions of recombination. (B) Inaccuracy in estimated positions of recombination.

In total, 46,520 recombinations were simulated over the first 333 cM in the nine simulations. We found switches in line origin probabilities for 88% of these using triM and 53% using GridQTL. Most of the recombination positions not found by GridQTL were in regions with missing probability estimates. GridQTL only identified 14 recombination events that were not found by triM. Imprecision in estimated recombination positions was nearly always higher for GridQTL than triM (Figure 5A), and inaccuracy was also higher on average for GridQTL (Figure 5B, Table 2). triM estimated more recombination positions with minimum imprecision than GridQTL and more with zero inaccuracy (Table 3).

**Table 2:** Differences between triM and GridQTL in estimating recombination positions

Method	Higher Imprecision	Higher Inaccuracy
triM	20	626
GridQTL	3502	2250

Results show the number of times one method performed less well than the other from 24,879 recombination positions that were found by both methods.

**Table 3:** Best estimates of recombination positions by triM and GridQTL

Method	Minimum Imprecision	Zero Inaccuracy
triM	1081	9318
GridQTL	693	8398

Results show the number of recombination positions estimated with the lowest possible imprecision and inaccuracy out of 24,879 cases that were found by both methods. The minimum imprecision is 1 cM.

**Figure 6:**
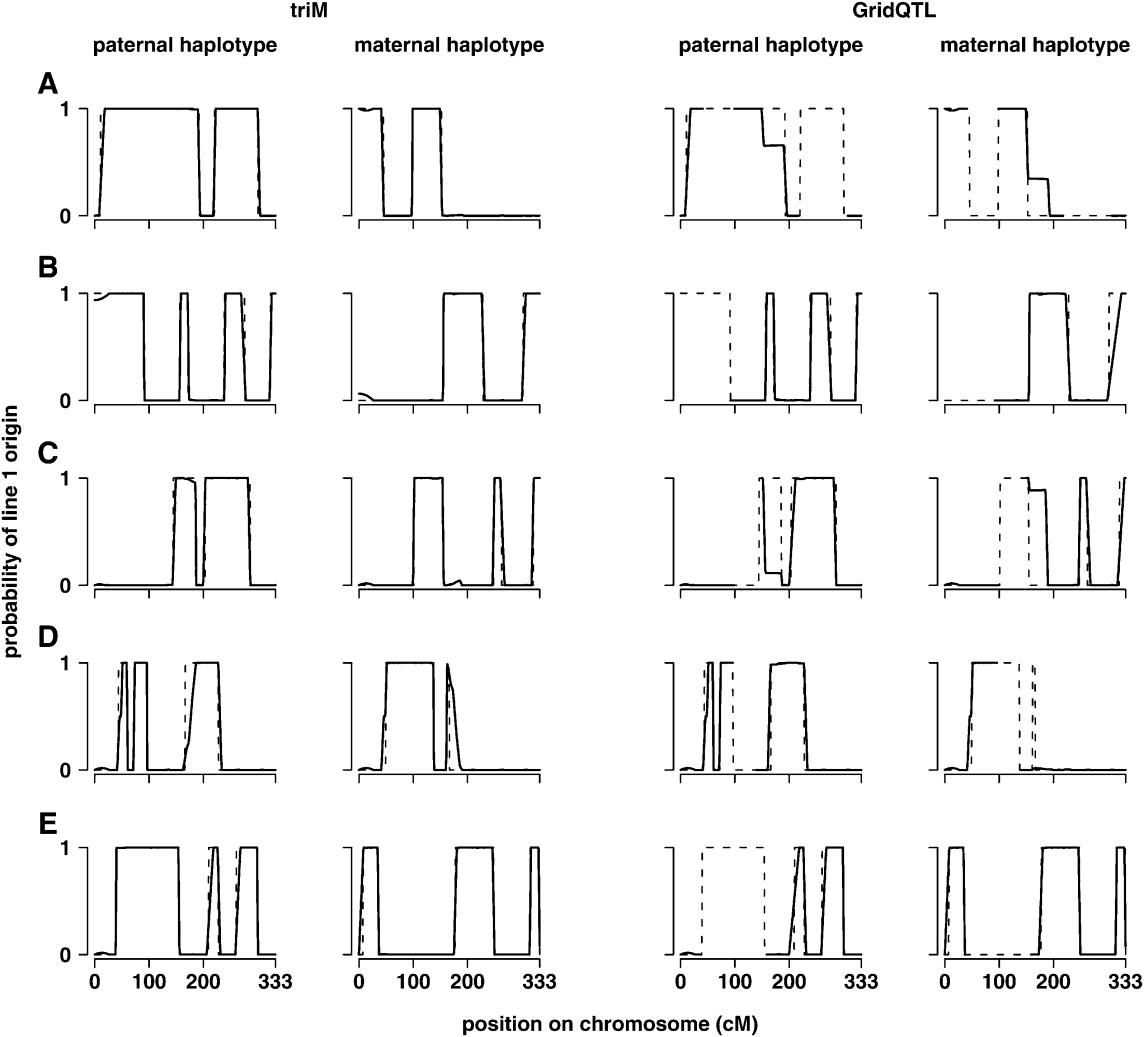
Examples of haplotype line origin probabilities. Estimated probabilities that the paternal and maternal haplotypes originate from line 1 are illustrated for examples where there were large differences between triM and GridQTL in estimating recombination positions. The left two graphs show results from triM, and the right two graphs show results from GridQTL. The paternal haplotype is shown on the left, and the maternal haplotype is shown on the right. Dashed lines show the simulated line origins. (A) The two cases where GridQTL was most imprecise in estimating the recombination position in comparison to triM; the simulated recombination points were at 192 cM in the father and 152 cM in the mother. (B) Another case where GridQTL had much higher imprecision than triM; the simulated recombination point was at 303 cM in the mother. (C) The two cases where GridQTL was most inaccurate in estimating the recombination position in comparison to triM; the simulated recombination points were at 186 cM in the father and 155 cM in the mother. (D) The case where triM was most imprecise and inaccurate in estimating the recombination position in comparison to GridQTL; the simulated recombination position was at 166 cM in the father. (E) One of the cases where triM was most inaccurate in estimating the recombination position in comparison to GridQTL; the simulated recombination position was at 210 cM in the father.

Examples of cases where triM and GridQTL substantially differed in estimating recombination positions are given in Figure 6. In the few cases where the imprecision was much higher for triM than GridQTL, it was often when there were recombinations from line 1 to 2 in one parent and line 2 to 1 in the other, within a few cM, and although GridQTL was less imprecise in the estimate of one recombination, it failed to detect the other at all (Figure 6D). In contrast, cases where imprecision was much higher for GridQTL seemed to be when there were recombinations in the same direction in both parents (*i.e.* both line 1 to line 2 or vice versa), around 40 cM apart (Figure 6A and C). There were other times when the parental line origin probabilities estimated by GridQTL changed more slowly than the estimates from triM (Figure 6B). When inaccuracy was higher for triM than GridQTL by more than 2 cM, either imprecision was also higher for triM (Figure 6D), or triM was less imprecise, so that the number of surrounding positions with a high line origin error was lower for triM (Figure 6E). Cases where GridQTL estimates were much more inaccurate tended to be cases where they were also more imprecise (Figure 6C).

## Discussion

Our study clearly illustrates the need for new algorithms to calculate line origin probabilities in outbred line cross experiments. The algorithm that is the basis of the most commonly used methods today (Haley *et al*., 1994) was not designed to handle dense SNP marker datasets and cannot be used for these in its original formulation. The GridQTL project (Seaton *et al.* 2006) provides a modified implementation of the Haley *et al.* (1994) algorithm that is reported to be useful for analyses of intermediate density SNP markers (up to 1500 markers per chromosome). Our comparisons however, illustrate that such extensions are not optimal, because for the GridQTL implementation (i) there are problems with the stability of the algorithm leading to either a significant portion of the analyses terminating before results are obtained or probabilities not being calculated for many individuals in large parts of the genome; (ii) the computational load, despite considerable computational resources, is approximately two orders of magnitude greater for intermediate size datasets and increases rapidly with the number of additional markers included in the analyses; and (iii) accuracy in estimates of line origin probabilities and inference of recombination positions is lower as a result of not using all the available genetic information. Although the stability of the algorithm might be resolved and it is possible that the time requirements could be somewhat improved, further development based on the Haley *et al.* (1994) algorithm is not recommended, as the algorithm is inherently less efficient than alternative algorithms and discarding marker information will inevitably give lower accuracy in estimates of line origin probabilities.

This study involved datasets of 1200-1500 markers, which was close to the maximum that could be handled by the modified Haley *et al.* (1994) algorithm implemented in GridQTL. This is, however, much lower than the number of markers that can currently be genotyped with SNP chips, for example, the chicken 60K SNP chip has approximately 8600 markers on chromosome 1. triM will be able to use all the marker information in such large datasets and complete the analyses rapidly.

A result that is of major concern for those attempting to analyze moderately dense SNP datasets with GridQTL is the large number of missing probability estimates; over 30% of the individuals had no estimates for the majority of the genome. Using GridQTL estimated probabilities rather than a complete set of probabilities to test for an association with phenotype will substantially reduce the power to detect QTL.

Data were simulated to closely reflect the pattern of marker information in real intercrosses. This was achieved by using founder haplotypes that were as near as possible to those in a well-studied chicken intercross and replicating the marker distances and pedigree structure. Therefore, the linkage disequilibria observed should closely resemble those that can be seen in real data. A series of replicates were created to ensure that findings were not due to peculiarities in specific runs; results showed relatively little variation across replicates. Hence, we believe that the findings in this study are representative of other large datasets.

triM was able to analyze the full set of markers and all replicates and give probability estimates for every position. Theoretically, the number of calculations performed by triM scales logarithmically with the number of markers, not exponentially as in the Haley *et al.* (1994) algorithm. This manageable increase in memory required means that triM should be capable of analyzing much larger marker datasets than those simulated here, including those that might become available in the future as SNP genotyping technology develops. There is no need for extensive preprocessing of the data; all the marker genotypes can be submitted so that no information is lost. In this study, triM was shown to be considerably faster than the modified Haley *et al.* (1994) algorithm implemented in GridQTL, taking seconds to complete the analyses rather than hours. The gain in speed was at least a factor of 80. The online documentation for GridQTL gives an estimated runtime of 48 hr for data from 1500 markers on one chromosome in 1000 individuals. As with memory, the time required by triM should increase moderately as more markers are included, so that data from large SNP chips could still be completed in a reasonable time.

Our simulations show that the average line origin error is low, around 2%, for both methods. The error was 10% larger for the GridQTL algorithm than for triM. Although the difference is small in absolute terms, triM did perform better and therefore should be preferred. It should also be noted that the observed difference in accuracy probably underestimates the true difference between the methods because only values that were estimated by GridQTL were compared. The points that GridQTL failed to estimate presumably included those where there were the longest stretches of partially informative markers, and therefore where the difference between the two methods could be greatest.

To gain a more detailed understanding of the differences between the two methods, we explored how well they inferred positions of recombination in the parents using measures of imprecision and inaccuracy. High imprecision or inaccuracy in estimating recombination positions is likely to explain most of the largest errors in line origin. GridQTL was nearly always more imprecise in estimating recombination positions than triM. There was less difference between the methods in the accuracy of the estimated positions, although GridQTL was more inaccurate than triM considerably more times than the other way round.

There is no detailed documentation of the changes made to the Haley *et al.* (1994) algorithm in GridQTL. It is likely that it uses a similar algorithm to that of QTL Express, the predecessor of GridQTL, where partially informative markers were discarded if necessary, so that there were no more than 15 partially informative markers in a stretch without intervening fully informative markers. Although these details are not available, we do not feel this affects our conclusions, as the differences between GridQTL and triM are in line with theoretical expectations.

We evaluated triM on a simulated F_2_ population. However, triM can equally be used for backcross populations or full or half sib family analyses. The codebase in which triM is implemented, cnF2freq, has additional capabilities. First, the grandparental chromosome that a region originates from can be estimated. This information can be used for more general analyses in a variance component framework without assuming QTL are fixed for alternate alleles in the two lines (Rönnegård *et al.* 2008). The functionality can also be extended into a full scheme for haplotyping. Inferring parental haplotypes can provide additional information on line origin by considering inheritance at linked markers. Second, different recombination rates can be applied for the two sexes. Although we simulated the same recombination rate in both sexes, sex differences in recombination rates of autosomes have been found in chicken (Kerje *et al.* 2003, Groenen *et al.* 2009) and other species. Use of the appropriate recombination rates for each sex should improve the probability estimates in general and is essential for correct analysis of the sex chromosomes. When the physical location or ordering of markers is known, a maximum likelihood fit of sex-specific recombination rates can also be computed, where the sex-averaged distances can be used as a starting value. Third, joint line origin probabilities, required for fitting models of more than one locus per chromosome (for example, in testing for epistasis) can be estimated. The line origin probabilities at two such positions are not independent if there is no fully informative marker between them and these dependencies have been included.

In conclusion, we have validated that triM provides accurate line origin probabilities from large SNP datasets and is extremely fast. A variant of the Haley *et al.* (1994) algorithm that has most commonly been used for this purpose was shown to be inferior for such data. triM was faster, more stable, provided more accurate line origin probabilities, and was better at inferring recombination positions. triM is therefore recommended as the method of choice for estimating line origin probabilities in outbred line crosses. triM is under continued development, and several useful additional functionalities are already available that will enable QTL analyses to keep pace with advances in genotyping technology.
